# Supplementary material for: Choosing between city and suburb: How urbanization shapes graduates’ housing preferences
Source: PLoS One. 2025 Nov 13;20(11):e0335960. doi: 10.1371/journal.pone.0335960 (PMC12614537; doi:10.1371/journal.pone.0335960)
Supplement: S1 File — This file contains the complete survey instrument used in this study, including measurement scales for employment opportunities, transportation convenience, living cost, work environment, satisfaction, housing choice, family economic support, and personal career planning. (DOCX) [file pone.0335960.s001.docx]

**Research Instrument Description**

This questionnaire is adapted from multiple validated sources:

Employment opportunity items derived from the Urban Employment Survey (UES, 2020)

Transportation convenience scale based on the China Urban Transport Assessment (CUTA, 2019)

Living cost measures adapted from the China Household Finance Survey (CHFS, 2021)

Housing choice items developed from the National Housing Preference Study (NHPS, 2022)

**Key Variable Definitions:**

1. Housing Choice: Measured as preference for urban center (rent/buy) vs. outskirts (rent/buy), with consideration of cost-effectiveness and diversity of options
2. Employment Opportunities: Assessed through availability, relevance to major, ideal job match, and career prospects
3. Transportation Convenience: Evaluated based on public transport availability, network development, commute time, and options variety

Dear interviewee:

We are conducting a study on the trade-off effect between employment opportunities and cost of living in urban centers and edges, aimed at understanding the factors and their impacts that undergraduate graduates consider when choosing their place of residence and workplace. Your valuable feedback will provide important assistance to our research. This questionnaire is anonymous, and all information is for academic research purposes only. Please feel free to fill it out. Completing this questionnaire will take approximately 10-15 minutes. Thank you for your support and participation!

**Part1 Basic information**

1. Your gender:

A. Male

B. Female

2. Your age:

A. 20-22 years old

B. 23-25 years old

C. 26-28 years old

D. Above 28 years old

3. Your current employment status:

A. Employed

B. Unemployed, actively seeking work

C. Continuing further education

D. Other

4. Your City：

A. Beijing

B. Shanghai

C. Guangzhou

D. Shenzhen

E. Chengdu

5. Housing Choice

I. My preferred housing arrangement is: (select one)

A. Renting in city center

B. Buying in city center

C. Renting in outskirts

D. Buying in outskirts

E. Other (please specify): _____

II. The most important factor in my housing choice is: (select top two)

A. Commute time to work

B. Housing cost

C. Neighborhood safety

D. Proximity to amenities

E. Future investment value

F. Space/size of housing

**Part 2 Main part (please mark a "√" in the corresponding column based on your situation)**

Note: please, indicate your opinions about each of the questions below by ticking one of the five responses provided in the column on the right side. The response scale ranges from “Strongly disagree” to “Strongly agree”. You may choose any of the five possible responses, as each represents a degree on the continuum.

| **Construct** | **Items** | 5  SA | 4  A | 3  N | 2  D | 1  SD |
| --- | --- | --- | --- | --- | --- | --- |
| \| **Employment Opportunities** \| 1. The city center offers more job opportunities. 2. The job opportunities in the city center are more in line with my major. 3. I am more likely to find my ideal job in the city center. 4. The career development prospects are better in the city center. 5. The job opportunities in the city outskirts are limited. \|  \| \| --- \| --- \| --- \| \| **Transportation Convenience** \| 1. Public transportation is more convenient in the city center. 2. The transportation network is more developed in the city center. 3. The commute from the city outskirts to the city center is longer. 4. There are fewer public transportation options in the city outskirts. 5. Traveling in the city center saves more time. \|  \| \| **Living**  **Cost** \| 1. The cost of living (such as rent, food, etc.) is higher in the city center. 2. The cost of living is relatively lower in the city outskirts. 3. Housing costs are my major expense in the city center. 4. The price levels are more reasonable in the city outskirts. 5. Living in the city center puts greater financial pressure on me. \|  \| \| **Work Environment** \| 1. The work facilities are more complete in the city center. 2. The corporate culture is stronger in the city center. 3. Collaboration among colleagues is closer in the city center. 4. The work environment is more relaxed in the city outskirts. 5. Work resources (such as information, technology, etc.) are more abundant in the city center. \|  \| \| **Satisfaction** \| 1. My overall satisfaction with the current place of residence. 2. My satisfaction with my current job. 3. My satisfaction with the transportation convenience of my residence. 4. My satisfaction with the employment opportunities in my residence. 5. My satisfaction with the housing options in my residence \|  \| \| **Housing Choice** \| 1. I prefer to rent in the city center. 2. I prefer to buy a house in the city outskirts. 3. There are more diverse housing options in the city center. 4. The cost-effectiveness of housing is higher in the city outskirts. \|  \| \| **Family Economic Support** \| 1. My family can support my living expenses in the city center. 2. Family economic support has a significant impact on my choice of residence. 3. My family can support my plan to buy a house in the city outskirts. 4. Family economic support makes it more likely for me to choose a job in the city center. \|  \| \| **Personal Career Planning** \| 1. My career planning requires me to work in the city center. 2. I am willing to accept the high cost of living in the city center for career development. 3. My career planning values the completeness of the work environment more. 4. I am willing to work in the city outskirts to balance the cost of living and job opportunities. 5. My long-term career planning is greatly influenced by the choice of residence. \|  \| | | | | | | |

Supplementary Notes:

1. Housing Choice Measurement:

Assessed through 4 dimensions:

Preference type (rent/buy)

Location (center/outskirts)

Diversity of options

Cost-effectiveness

Rated on 5-point Likert scale from "Strongly Disagree" to "Strongly Agree"

2. Urban Center Definition:

Primary business districts with dense employment opportunities

Typically within 5km radius of city's geographical center

3. City Outskirts Definition:

Suburban areas beyond 10km from city center

Characterized by lower density and more residential focus

**References**

China Household Finance Survey. (2021). Housing preference module. Southwestern University of Finance and Economics.

National Housing Preference Study. (2022). Urban-suburban housing choice measures. China Academy of Urban Planning and Design.

Urban Employment Survey. (2020). Employment opportunity assessment scale. Peking University Institute of Social Science Survey.

China Urban Transport Assessment. (2019). Public transportation convenience index. Ministry of Transport of China.
