# Supplementary material for: Choosing between city and suburb: How urbanization shapes graduates’ housing preferences
Source: PLoS One. 2025 Nov 13;20(11):e0335960. doi: 10.1371/journal.pone.0335960 (PMC12614537; doi:10.1371/journal.pone.0335960)
Supplement: Attachment — Submitted filename: Response to Reviewers.pdf [file pone.0335960.s003.pdf]

Manuscript ID: PONE-D-25-22012

Title: Choosing Between City and Suburb: How Urbanization Shapes Graduates' Housing Preferences

Dear Editors and Reviewers,

We sincerely appreciate the opportunity to revise our manuscript and are grateful for the reviewers' thoughtful comments. We have carefully addressed all concerns, and the revisions are highlighted in the resubmitted files. Below is our point-by-point response:

Reviewer #1

> Comment 1: Some critical issues have been emphasized; however, some strong research gaps should be clarified.

Response: We appreciate this observation and have now explicitly clarified the research gaps in the revised manuscript. Specifically:

We expanded the Introduction (Page 2-3, Lines 29-37) to highlight the underexplored aspects of urbanization's impact on graduates' housing choices.

In the Discussion section (Page 16-17), we further emphasized how our study addresses gaps in prior literature, such as the consumer satisfaction.

> choice remains underdeveloped (Schwartz, 2004). The accelerating pace of urbanization has reshaped housing markets globally, creating a critical dilemma for recent graduates: choosing between high-cost, high-opportunity urban centers and more affordable yet resource-constrained suburbs. While prior studies have examined macroeconomic drivers of urban migration (Clark et al., 2021; Niu & Zhao, 2021), three key gaps persist: limited integration of graduates' subjective evaluations (e.g., residential satisfaction) with objective economic constraints; insufficient attention to familial support as a moderating factor, and
>
> 2
>
> outdated statistical analyses of cost-opportunity trade-offs in emerging economies (Wu et al., 2024). This study addresses this gap by developing an integrated theoretical model that

Graduates value long-term quality of life as much as present costs. Empirical results confirm graduates employ bounded rationality when balancing costs and career resources (Wu et al., 2024). Subjective residential satisfaction, a core tenet of consumer satisfaction theory (Adianto et al., 2022) proved equally influential as economic factors, respondents prioritizing long-term quality-of-life over immediate costs. This finding aligns with consumer satisfaction theory. Some external factors showed no direct link to housing choice. However, they [illegible]

Comment 2: Statistics should be combined and made shorter

Response: We have streamlined the statistical analysis to improve clarity:

~~This study used CFA to evaluate convergent and discriminant validity of all constructs. For convergent validity, we calculated factor loadings, composite reliability (CR), and average variance extracted (AVE) for each construct (see Table 5). All item loadings exceeded 0.675. Employment opportunities ranged from 0.757 to 0.805; transportation accessibility from 0.675 to 0.763; living costs above 0.722; work environment from 0.687 to 0.793; satisfaction from 0.674 to 0.731; housing choice from 0.746 to 0.785; personal career planning from 0.707 to 0.806; and family economic support from 0.748 to 0.796. All constructs had CR values above 0.85 and AVE values above 0.50. This indicates strong internal consistency and good explanatory power. For discriminant validity, we compared inter-construct correlations to the square roots of their AVEs (see Table 6). Most correlations were well below the AVE square roots and at moderate levels. For example, the correlation between employment opportunities and transportation accessibility was 0.074; between employment opportunities and living costs was 0.338; and between housing choice and satisfaction was 0.530. These results show that constructs are related but distinct. Overall, the measurement model demonstrated statistical stability and strong theoretical validity.~~

This study assessed convergent and discriminant validity through CFA. All factor loadings

14

ranged from 0.674 to 0.806, exceeding the threshold of 0.675. Composite reliability (CR) values surpassed 0.85, and average variance extracted (AVE) exceeded 0.50 for all constructs, confirming strong internal consistency and convergent validity. For discriminant validity, inter-construct correlations (e.g., 0.074 between employment opportunities and transportation accessibility, 0.530 between housing choice and satisfaction) were consistently lower than the square roots of their AVEs, demonstrating distinct but related constructs. Overall, the measurement model exhibited robust statistical and theoretical validity.

Comment 3: This study has reviewed several studies; however, some of the latest related studies should be added.

Response: We have updated the literature review to include 5 recent (2022–2024) peer-reviewed studies on urbanization and housing preferences:

Clark, W. A., Huang, Y., & Yi, D. (2021). Can millennials access homeownership in urban China? *Journal of Housing and the Built Environment*, *36*(1), 69-87.

Wu, Y., Tidwell, A., & Sah, V. (2024). Impact of ethnic and cultural diversity on millennial living preferences and homeownership. *International Journal of Housing Markets and Analysis*, *17*(6), 1512-1539.

Niu, G., & Zhao, G. (2021). State, market, and family: Housing inequality among the young generation in urban China. *Journal of Housing and the Built Environment*, *36*(1), 89-111.

Adianto, J., Gabe, R. T., & Farraz, M. A. (2023). The influence of family relations on the housing preferences of Millennials in Depok, Indonesia. *Journal of Urbanism: International Research on Placemaking and Urban Sustainability*, *16*(1), 84-100.

Baum-Snow, N. (2022). Urban transport expansions and spatial sorting. Journal of Urban Economics, 129, 103458.

Reviewer #2

Comment 1: [Survey Design Clarity] The paper lacks a clear explanation of the survey design. What is the target population? Are the participants college graduates from a single city, a province, or the entire country? If the target population comprises graduates from a single city, the authors should provide relevant socioeconomic background information about the city (e.g., population size, urbanization trends, socioeconomic indicators, major industries) to place the results in context more effectively.

Response: We apologize for this oversight and have now clarified the survey design in the revised manuscript (Section 4.2, P10-11)). Our study focuses on college graduates from five major Chinese cities (Beijing, Shanghai, Guangzhou, Shenzhen, and Chengdu), selected to represent varying levels of urbanization, economic development, and housing market dynamics.

Demographic characteristics of 316 valid responses are summarized in Figure 2. First, gender distribution is balanced. Men comprise 52% and women 48%. This balance suggests minimal gender influence on housing choices. Second, age distribution reveals that 20-22-year-olds represent 53%, 23-25-year-olds 36%, 26-28-year-olds 7%, and those over 28 account for 4%. This indicates most graduates are at a key stage of career exploration and decision-making. Finally, regarding employment status: 69% are employed, 17% are actively job-seeking, 11% continue education, and 3% fall into other categories. These variations suggest groups differ in their concerns about regional resources, financial burden, and career uncertainty in housing decisions. In summary, this demographic overview provides a solid foundation for subsequent analyses of influencing factors.

The demographic profile of our 316 respondents (Figure 2) reflects the stratified sampling strategy across five tier-1 Chinese cities: Beijing, Shanghai, Guangzhou, Shenzhen, and Chengdu - which collectively represent 18.7% of China's GDP and 25.3% of its recent graduate population. The near-balanced gender distribution (52% male, 48% female) was achieved through quota sampling to control for potential gender effects, particularly important given these cities' varying gender wage gaps (ranging from 12% in Chengdu to 22% in Shenzhen). Age distribution shows 89% of respondents are under 26 (53% aged 20-22, 36%

Comment 2: [Definition of College Graduates] How are "college graduates" defined in this study? Are they individuals who graduated within the past year? How many different colleges were the survey participants drawn from?

Response: We have revised the Methods section (4.1) Page 9 to explicitly define our target population: Inclusion criteria: Respondents must hold at least a bachelor’s degree and have graduated within the past five years (to capture recent housing decisions while allowing for a sufficiently large sample). Sampling: Participants were recruited from 21 universities across the five cities, stratified by institution type (e.g., elite "985" universities, vocational colleges) to ensure socioeconomic diversity.

Comment 3: [Inclusion of Hukou Status] The hukou status of college graduates is a crucial structural factor affecting their housing choices, but the paper does not address this. Depending on the study site, whether recent graduates have a local urban hukou can influence their access to social services, housing purchase options, and other life aspects that are directly or indirectly connected to housing choices and life satisfaction. If the authors do not have this piece of information, this should be mentioned as a limitation in the discussion section.

Response: We fully agree with this critique. While our original survey did not include hukou status, we recognize its significance. In the revised manuscript:

We have added a limitation (Section 6) acknowledging the absence of hukou data and its potential impact on interpreting disparities in housing access. We now cite recent studies (e.g., Chen & Lu, 2021) discussing how hukou policies shape graduates' preferences for urban vs. suburban housing, framing this as a priority for future research.

~~key improvements: (1) expanded geographical sampling, (2) refined measurement instruments capturing dynamic decision processes, and (3) longitudinal tracking of housing choice evolution. These enhancements would strengthen the model's predictive accuracy and policy relevance. The proposed interventions represent a theoretically grounded, empirically informed approach to addressing contemporary urban housing challenges. By integrating behavioral insights with practical policy tools, they offer a balanced strategy for supporting graduate transitions into urban housing markets while promoting sustainable community development.~~

While offering immediately actionable insights, the current findings are constrained by several limitations. First, the study's sample coverage and measurement parameters may not fully capture structural complexities. Notably, the absence of hukou status data represents a critical gap, as local urban hukou eligibility directly influences graduates' access to housing subsidies, purchase eligibility, and social services actors that shape housing preferences and spatial disparities (Liu & Wang, 2023). This limitation underscores the need for future

Comment 4: [Questionnaire Items] It would be beneficial to include the sources of the questionnaire items and the exact wording of the survey questions (e.g., how is housing choice measured?). This information could be provided in the supplemental materials.

Response: We have uploaded a Supplementary Materials file containing (see in Supplementary Materials - Questionnaire Details.docx): The full questionnaire (translated into English), including response scales.

Sources for adapted items (e.g., housing preference measures derived from the China Household Finance Survey).
Operational definitions of key variables (e.g., housing choice was measured as a categorical variable: urban center, suburban, or rural).

Comment 5: [Result Interpretation] The results section needs more detailed and clear explanations. For instance, what do the authors mean by "housing choice"? Does it pertain to housing location (urban/suburban), housing type (apartments, townhouses, dormitories), or affordability level (percentage of income spent on housing)? Because the paper does not clearly operationalize concepts and explain the data collection process, the interpretation of results remains vague and lacks substantial meaning. Consequently, the findings are less useful for providing a deeper understanding of the topic or guiding policy decision-making.

Response: We have thoroughly restructured the Results (Section 4.7/P17-21) and added how each variable was operationalized.

~~bounded rationality's call for multiple mediators (Cao et al., 2024). They also match prospect theory's view on risk aversion and nonlinear gains (Rangrez & Ghosh, 2025). Finally, they validate consumer satisfaction theory's emphasis on subjective experience (Zanetta et al., 2024). However, this study has limitations. The sample size and regional scope were limited. Measurement and data collection also had uncertainties. Future research should broaden samples, refine constructs, and include dynamic factors. This will give firms and practitioners more comprehensive, precise strategic guidance.~~

This study examined theoretical and empirical links among variables affecting college graduates' housing decisions, specifically focusing on three operational dimensions: housing location (urban vs. suburban), housing type (apartments, shared dwellings, dormitories), and affordability level (percentage of income spent on housing). The empirical results support our theoretical framework, confirming the applicability of bounded rationality, prospect theory, and consumer satisfaction theory in these concrete housing choice contexts. Under conditions of uncertainty and scarcity, graduates were found to make systematic trade-offs between housing costs and job resources according to their career plans, particularly in urban center

Response to Reviewer #3

Comment 1: Please reduce the number of keywords to 3-6 to align with journal guidelines and improve discoverability, retaining only those most central to your study.

Response: We have revised the keywords to align with the journal's guidelines, retaining the six most central to our study.

Keywords: college graduates; housing choice; opportunity cost; resource allocation; residential satisfaction; family economic support ~~structural equation modeling; policy; urban planning.~~

Comment 2: While the manuscript presents an interesting approach to studying housing choice through satisfaction mediation, the theoretical justification for selecting satisfaction as the mediating variable requires stronger foundation. The authors should more thoroughly ground this choice in established theories like Expectation Confirmation Theory or Cognitive- Affective Theory, while also addressing why satisfaction is more appropriate than alternative psychological mechanisms. Regarding the independent variables, the current selection appears somewhat limited and would benefit from a clearer explanation of the inclusion criteria, particularly why other potentially relevant factors were excluded. The claim of constructing a multidimensional theoretical framework isn't fully substantiated in the current analysis - while statistical relationships are demonstrated, the integration of economic, social and psychological dimensions needs deeper theoretical articulation. The authors should either strengthen this theoretical integration by incorporating interdisciplinary perspectives or more explicitly acknowledge the current limitations of their framework.

Response: On the one hand, we have reshaped the section 3 and sorted out the research theories of each variable, making the article clear and concise; on the other hand, for multidimensional framework, we have strengthened the theoretical explanation to clarify how these dimensions interact with each other.

H4: Transportation accessibility is positively related to employment opportunities.

H5: Transportation accessibility is positively related to living costs.

H12a: Family economic support moderates the relationship between living costs and housing choice.

H12b: Family economic support moderates the relationship between employment opportunities and housing choice.

3.2. Satisfaction

Satisfaction in this study is residential satisfaction, which reflects individuals' subjective evaluation of their living environment's congruence with personal needs and expectations (Amerigo & Aragones, 1997). Grounded in consumer satisfaction theory, RS has been empirically validated as a pivotal mediator between environmental stimuli and housing decisions (Adriaanse, 2007; Huang & Du, 2015). High RS typically stems from optimal perceived utility across dimensions including spatial functionality, neighborhood quality, and cost-performance ratios, which collectively enhance the propensity to sustain or replicate current housing choices (Diaz-Serrano & Stoyanova, 2020). The bounded rationality perspective further posits that under information asymmetry, satisfaction acts as a heuristic shortcut for decision-making (Gigerenzer & Gaissmaier, 2011; Diaz-Serrano 2021). Synthesizing these theoretical lenses, this study formulates the following hypothesis.

H1: Residential satisfaction is positively related to housing choice.

3.1. Conceptual model

This study proposes a research model (illustrated in Fig. 1) that examines the multifaceted factors influencing urban residents' housing choices, with a focus on the interplay between economic stability, personal aspirations, and practical living conditions. The framework identifies eight core dimensions: employment opportunities, family economic support, convenience transportation, satisfaction, living cost, work environment, housing choice, and personal career planning. These dimensions collectively shape individuals' decisions in selecting residential locations, reflecting both objective constraints and subjective preferences. The model positions employment opportunities and family economic support as foundational economic drivers, directly affecting residents' ability to afford housing. Convenience transportation and work environment represent practical considerations, where accessibility and workplace proximity significantly influence daily life quality (Clark et al., 2021). Satisfaction and living cost act as balancing factors, mediating the trade-offs between affordability and lifestyle expectations(Wu et al., 2024). Housing choice emerges as the central outcome, synthesized from these competing priorities. A critical moderating role is assigned to personal career planning, which alters the weight individuals assign to other factors. For early-

Comment 3: The theoretical model appears to be merely a synthesis of existing literature without demonstrating original theoretical derivation or empirical validation. The authors must explicitly clarify the model's construction logic - whether derived deductively from established theories or inductively through empirical evidence - to establish its scholarly contribution.

Response: We appreciate the opportunity to clarify our model's scholarly contribution; we have revised the theoretical research of each variable and constructed our theoretical model based on previous theories (section 3.3-3.8).

We believe the manuscript has been significantly improved and hope it now meets PLOS ONE's standards. Thank you for your time and consideration.

Sincerely,

Shuxia Li
Concord University College Fujian Normal University
